# Accuracy of epidemiological inferences based on publicly available information: retrospective comparative analysis of line lists of human cases infected with influenza A(H7N9) in China

**DOI:** 10.1186/1741-7015-12-88

**Published:** 2014-05-28

**Authors:** Eric HY Lau, Jiandong Zheng, Tim K Tsang, Qiaohong Liao, Bryan Lewis, John S Brownstein, Sharon Sanders, Jessica Y Wong, Sumiko R Mekaru, Caitlin Rivers, Peng Wu, Hui Jiang, Yu Li, Jianxing Yu, Qian Zhang, Zhaorui Chang, Fengfeng Liu, Zhibin Peng, Gabriel M Leung, Luzhao Feng, Benjamin J Cowling, Hongjie Yu

**Affiliations:** 1 School of Public Health, Li Ka Shing Faculty of Medicine, The University of Hong Kong, Hong Kong, Special Administrative Region, China; 2 Division of Infectious Disease, Key Laboratory of Surveillance and Early-warning on Infectious Disease, Chinese Center for Disease Control and Prevention, Beijing, China; 3 Network Dynamics and Simulation Science Laboratory, Virginia Bioinformatics Institute, Virginia Tech, Blacksburg, VA, USA; 4 Informatics Program, Boston Children’s Hospital, Boston, MA, USA; 5 Department of Pediatrics, Harvard Medical School, Boston, MA, USA; 6 FluTrackers International Charity, Florida 32789, USA

**Keywords:** Epidemiological monitoring, Line list, Infectious disease outbreak, Influenza A virus, H7N9 subtype

## Abstract

**Background:**

Appropriate public health responses to infectious disease threats should be based on best-available evidence, which requires timely reliable data for appropriate analysis. During the early stages of epidemics, analysis of ‘line lists’ with detailed information on laboratory-confirmed cases can provide important insights into the epidemiology of a specific disease. The objective of the present study was to investigate the extent to which reliable epidemiologic inferences could be made from publicly-available epidemiologic data of human infection with influenza A(H7N9) virus.

**Methods:**

We collated and compared six different line lists of laboratory-confirmed human cases of influenza A(H7N9) virus infection in the 2013 outbreak in China, including the official line list constructed by the Chinese Center for Disease Control and Prevention plus five other line lists by HealthMap, Virginia Tech, Bloomberg News, the University of Hong Kong and FluTrackers, based on publicly-available information. We characterized clinical severity and transmissibility of the outbreak, using line lists available at specific dates to estimate epidemiologic parameters, to replicate real-time inferences on the hospitalization fatality risk, and the impact of live poultry market closure.

**Results:**

Demographic information was mostly complete (less than 10% missing for all variables) in different line lists, but there were more missing data on dates of hospitalization, discharge and health status (more than 10% missing for each variable). The estimated onset to hospitalization distributions were similar (median ranged from 4.6 to 5.6 days) for all line lists. Hospital fatality risk was consistently around 20% in the early phase of the epidemic for all line lists and approached the final estimate of 35% afterwards for the official line list only. Most of the line lists estimated >90% reduction in incidence rates after live poultry market closures in Shanghai, Nanjing and Hangzhou.

**Conclusions:**

We demonstrated that analysis of publicly-available data on H7N9 permitted reliable assessment of transmissibility and geographical dispersion, while assessment of clinical severity was less straightforward. Our results highlight the potential value in constructing a minimum dataset with standardized format and definition, and regular updates of patient status. Such an approach could be particularly useful for diseases that spread across multiple countries.

## Background

Emerging and re-emerging infectious diseases pose a continuing threat to human health. In the past decade we have faced global epidemics including SARS-coronavirus, pandemic influenza A(H1N1)pdm09 virus, avian influenza A(H5N1) virus, and most recently we have witnessed the emergence of influenza A(H7N9) virus in China, and the Middle-East-Respiratory-Syndrome (MERS)-coronavirus in the Middle East and Europe. Appropriate public health responses to infectious disease threats should be based on the best-available evidence, which in turn requires reliable data and appropriate analysis. In particular, risk assessments for A(H7N9) and MERS-coronavirus involve estimation and characterization of transmissibility and clinical severity [1-3].

Provided incidence of laboratory-confirmed cases is low, it is possible for health authorities to collect detailed data on each confirmed case in a ‘line list’. Analysis of this information can provide important insights into the epidemiology of a specific disease. A notable aspect of the recent epidemics of A(H7N9) and MERS-coronavirus is the amount of information about individual cases provided online, through official press releases and various media sources, to a much greater extent than, for example, during the A(H1N1) pandemic in 2009 to 2010 and the Severe Acute Respiratory Syndrome epidemic in 2003.

The influenza A(H7N9) virus emerged in early 2013 in China, and 143 laboratory-confirmed cases had been reported in mainland China by the end of 2013, with the majority of confirmed cases having illness onset during March and April 2013 [4]. The Chinese National Health and Family Planning Commission notified the World Health Organization in late March and joined forces for the prevention and control of the disease, along with other international animal health organizations [5]. Initiatives such as The Global Initiative on Sharing All Influenza Data (GISAID) have provided a framework for the sharing of full sequence data on virus genomes [6]. In the A(H7N9) epidemic, GISAID fostered several studies in early April, such as comparison of the A(H7N9) virus against Eurasian avian influenza viruses [7] and avian influenza A(H7N7) in the Netherlands [8]. There is no similar framework for the sharing of epidemiological data, although a number of unofficial line lists and repositories of epidemiologic data have been created based on publicly available data by automated digital surveillance algorithms or epidemiologists [9]. The objective of the present study was to investigate the extent to which reliable epidemiologic inferences could be made based on publicly available epidemiologic data, compared to the official data collected by Chinese health authorities on laboratory-confirmed cases of influenza A(H7N9) virus infection.

## Methods

### Ethical approval

It was determined by the Chinese National Health and Family Planning Commission that the collection of data from influenza A(H7N9) cases was part of a continuing public health investigation of an emerging outbreak and was exempt from institutional review board assessment.

### Sources of data

A line list with detailed epidemiologic information on each laboratory-confirmed case of influenza A(H7N9) virus infection was constructed by the Chinese Center for Disease Control and Prevention (China CDC). Case definitions, surveillance for identification of A(H7N9) cases and A(H7N9) laboratory assays are described in a previous report [10]. Relevant epidemiological data on A(H7N9) cases were collected through interviews by trained staff. Data used in the present analyses include age, sex, geographic location (city and province), health status on admission, and dates of illness onset, hospital admission, death or discharge, for cases which were officially announced as of 31 May 2013, when the epidemic had stabilized.

In addition to the ‘official’ China CDC line list, we collated five other line lists that were constructed based on publicly available data. The five line lists were created by Harvard Medical School/Boston Children’s Hospital (‘HealthMap’), Virginia Polytechnic Institute and State University (‘Virginia Tech’), Bloomberg News (‘Bloomberg’), the University of Hong Kong School of Public Health (‘HKUSPH’), and FluTrackers [see Additional file 1: data file]. HealthMap is an automated disease surveillance system specializing in real-time geospatial visualization of disease outbreaks [11]. FluTrackers is an online forum which tracks and hosts discussions of a wide range of infectious diseases [12]. Virginia Bioinformatics Institute, Virginia Tech, and HKUSPH were staffed with a group of epidemiologists with interest in the modeling of infectious disease epidemics. Bloomberg news agency collated basic epidemiological data to assist with monitoring of the outbreak. Each line list was compiled based on reports of laboratory-confirmed influenza A(H7N9) cases released by, in the order of importance, the national and provincial Ministry of Health websites or microblogs, World Health Organization, international online disease reporting systems and online Chinese news or blogs [see Additional file 2: Table S1].

### Statistical methods

We first conducted descriptive comparisons of the accuracy of individual variables in each line list compared to the China CDC version on various dates. Then we used line lists available at specific dates to estimate key epidemiologic parameters including the distributions of time from illness onset to hospitalization delay, time from illness onset to death, and time from onset to discharge, without adjusting for right-censoring which would require regular updates on patient status. Finally, we used the line lists available at specific dates to replicate real-time inferences on the hospitalization fatality risk (HFR) and the impact of closure of live poultry markets. We analyzed the line lists starting from 10 April 2013, when the number of confirmed A(H7N9) cases surpassed 30, until 31 May 2013. As the line lists were updated independently at different dates, for comparison purpose the dates of analyses were chosen to match the time of updates for most line lists.

To study inferences on clinical severity, we estimated the HFR [3] at specific calendar dates using two approaches. First, we divided the cumulative number of deaths by the cumulative number of hospitalized cases (HFR_1_), an approach which is certain to underestimate the hospitalization fatality risk because unresolved cases destined to die are included in the denominator but not the numerator [13,14]. Second, we divided the cumulative number of deaths by the cumulative number of cases who had either died or been discharged (recovered). This approach (HFR_2_) should give an accurate real-time estimate of the HFR if the distribution of times from onset to death is similar to the distribution of times from onset to discharge, and the HFR does not change over calendar time [14].

To study inferences on transmissibility, we estimated the impact of closure of live poultry markets in Shanghai, Nanjing and Hangzhou using Poisson regression models that compared the incidence rates of confirmed A(H7N9) cases since the first case in each city versus the incidence rates after closures [15,16]. We allowed for incubating infections by excluding a two-day ‘washout’ period immediately after market closures, with other washout periods considered in sensitivity analyses. We used multiple imputation with 20 replications for missing dates of illness onset in each dataset, based on the empirical onset to reporting distribution [17,18]. All statistical analyses were conducted using R version 3.0.1 (R Foundation for Statistical Computing, Vienna, Austria).

## Results

Age, sex, province and date of illness and death were collected for each influenza A(H7N9) case in all six line lists (Table 1). Current health status was also collected but only the China CDC, Virginia Tech and FluTrackers line lists had more detailed information on severity. Information was updated daily for China CDC and HealthMap while other line lists had more frequent updates at the beginning of the epidemic and less frequent updates when the epidemic tapered in early May. FluTrackers also updated their line list daily but was able to retrieve historical archives for the specific dates as listed in Table 1. More than 90% of the cases could be matched to the China CDC line lists by age, sex, province and date of illness onset [see Additional file 3: Figure S1]. While information on age, sex and province were mostly complete in different line lists, there were significant proportions of missing data on dates of hospitalization, discharge and health status. Death and discharge dates that were only available weeks after illness onset had a greater proportion of missing information [see Additional file 3: Figure S2]. For matched cases, we found discrepancies in dates of hospitalization, death and discharge when comparing to the China CDC line list [see Additional file 3: Figure S3].

**Table 1:** Summary of epidemiological information collected in each line list

**Information collected for each case**	**China CDC**	**HealthMap**	**Virginia Tech**	**Bloomberg**	**HKU SPH**	**FluTrackers**
Age	×	×	×	×	×	×
Sex	×	×	×	×	×	×
Province	×	×	×	×	×	×
City	×	×	×			×
Date of illness onset	×	×	×	×	×	×
Date of hospital admission	×		×	×	×	×
Date of discharge	×			×	×	
Date of death	×	×	×	×	×	×
Health status	discharged/mild/stable/severe/died	died	discharged/mild/stable/severe/died	discharged/died	mild/severe/died	discharged/mild/stable/severe/died
Dates of data updates/archives	Apr 5 - May 31	Apr 5 - May 31	Apr 22, 24, 25, 26, 29, May 1, 6, 7	Apr 5–11, 13–19, 22–26, 29, 30, May 2, 7	Apr 10–24, May 1, 6, 13, 20, 27	Apr 13, 15, 16, 18, 22, 23, 27, 28, 30, May 2, 6, 9, 17, 29, 31

China CDC, Chinese Center for Disease Control and Prevention; HKUSPH, The University of Hong Kong School of Public Health.

**Figure 1:**
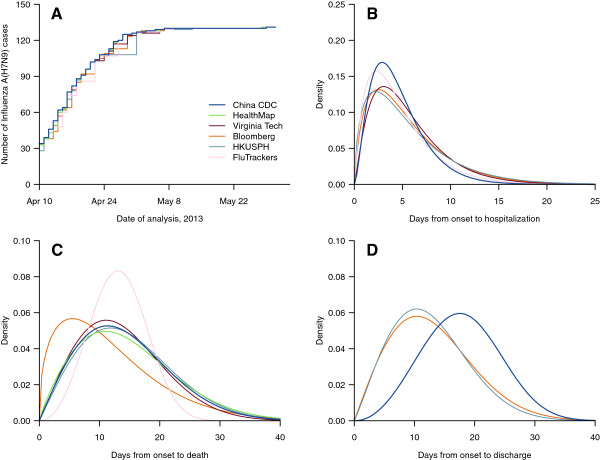
**Epidemiological distributions based on analysis of line lists on 1 May 2013. (A)** Number of laboratory-confirmed cases of influenza A(H7N9) virus infection, 10 April to 31 May, 2013. **(B)** onset-to-hospitalization distribution. **(C)** onset-to-death distribution. **(D)** onset-to-discharge distribution. Date of analysis refers to US local time for HealthMap, Virginia Tech and FluTrackers line lists, and China local time for China CDC, Bloomberg and HKUSPH line lists. China CDC, Chinese Center for Disease Control and Prevention; HKUSPH, the University of Hong Kong School of Public Health.

We compared different epidemiological characteristics inferred from different line lists over time, for all cases irrespective of matching. The reported number of cases from the five line lists followed closely those reported by the China CDC line list, with less than one-day time-lag (Figure 1). The epidemic curves from the HealthMap, HKUSPH, Virginia Tech and FluTrackers line lists also resembled that from the China CDC line list at different time points [see Additional file 3: Figure S4], although some of the onset dates were missing or inaccurate. We estimated the onset to hospitalization distribution by a Gamma distribution, and onset to death and discharge distribution by Weibull distribution [4]. The estimated onset to hospitalization distributions on 1 May 2013 were generally similar (median ranged from 4.6 to 5.6 days) for all line lists (Figure 1). HealthMap, HKUSPH and Virginia Tech line lists were able to reflect the longer onset to death period for patients staying longer in hospital [see Additional file 3: Figure S5]. Information on discharge dates was only available in the Bloomberg and HKUSPH line lists, and in those datasets the estimated onset to discharge distributions were much shorter than the distribution based on the China CDC line list, with more missing discharge dates at the end of April [see Additional file 3: Figures S2 and S5]. We were able to obtain robust estimates for the onset to hospitalization distribution from each of the line lists early in the epidemic, but robust estimates of the onset to death distribution were not available until early May [see Additional file 2: Table S2].

**Figure 2:**
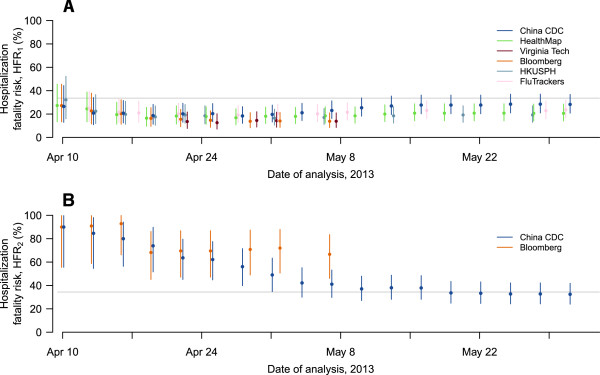
**Estimated hospitalization fatality risks for laboratory-confirmed Influenza A(H7N9) cases, 10 April to 31 May, 2013. (A)** HFR_1_ based on the number of deaths divided by the number of confirmed cases. **(B)** HFR_2_ based on the number of deaths divided by the number of confirmed cases with known outcome (death or discharge). HealthMap, Virginia Tech and HKUSPH did not routinely collect data on the number of discharged patients. The most updated estimate of the HFR [19] is shown by the gray lines. Vertical lines indicate the 95% confidence intervals. Date of analysis refers to US local time for HealthMap, Virginia Tech and FluTrackers line lists, and China local time for China CDC, Bloomberg and HKUSPH line lists. China CDC, Chinese Center for Disease Control and Prevention; HFR, hospitalization fatality risk; HKUSPH, the University of Hong Kong School of Public Health.

Figure 2 shows the estimated hospitalization fatality risk under the two different approaches. HFR_1_ estimates were consistently around 20% before May for all line lists and approached 35% afterwards. The five line lists consistently under-estimated HFR_1_ although the 95% confidence intervals covered the true estimate. As of 31 May, there were 18 patients with unresolved outcomes, including 16 patients with severe condition. The estimation of HFR_2_ required more detailed information (discharge status) and was only available for the China CDC and Bloomberg line lists. HFR_2_ decreased over time and stabilized at around 30% to 40% in early May. The Bloomberg estimates tended to be higher than the China CDC HFR_2_ with increasingly larger discrepancies over time. Only the HealthMap and FluTrackers line lists were able to provide more robust estimates of the fatality risk for hospitalized cases near the end of the study [see Additional file 2: Table S2].

**Figure 3:**
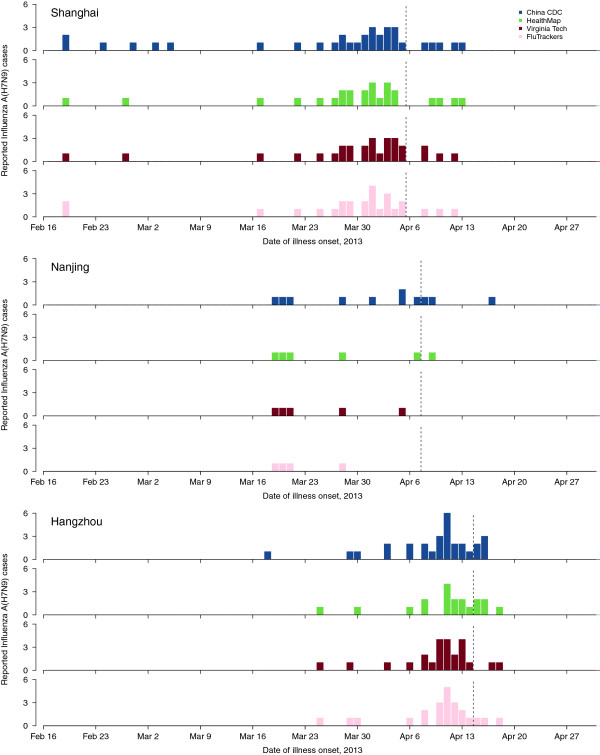
**Dates of illness onset of influenza A(H7N9) cases in Shanghai, Nanjing and Hangzhou.** Dotted lines show the dates of live poultry market closure in each city. Patients with missing onset dates were excluded.

The epidemic curves in Shanghai and Hangzhou were very similar based on the China CDC, HealthMap, Virginia Tech and FluTrackers line lists where information on geographic location was available to the city level (Figure 3), athough there were some missing onset dates [see Additional file 3: Figure S2]. Live poultry market closures were implemented on 6 April, 8 April and 15 April in Shanghai, Nanjing and Hangzhou, respectively. Except for the FluTrackers line list where all onset dates after April were not available in Nanjing, market closures in all three cities were consistently estimated to be extremely effective in reducing A(H7N9) incidence rates (Table 2).

**Table 2:** Estimated effect of live poultry market closure in Shanghai, Nanjing and Hangzhou

**Line list**	**Estimated incidence rate ratio (*P *** **value)**		
	**Shanghai**	**Nanjing**	**Hangzhou**
China CDC	0.064 (<0.001)	0.014 (0.007)	0.000 (<0.001^b^)
HealthMap^a^	0.067 (<0.001)	0.000 (0.034^b^)	0.007 (<0.001)
Virginia Tech^a^	0.037 (<0.001)	0.017 (0.010^b^)	0.006 (<0.001)
FluTrackers^a^	0.061 (<0.001)	0.362 (0.328)	0.004 (<0.001)

^a^Missing onset dates were imputed based on the empirical onset-to-report distribution from other A(H7N9) cases in Shanghai, Nanjing and Hangzhou. ^b^There were no cases two days after the market closure in Nanjing and Hangzhou. *P* values were based on likelihood ratio tests. China CDC, Chinese Center for Disease Control and Prevention.

## Discussion

We examined which important epidemiological inferences could be drawn from publicly available information compared to official data from China CDC. We demonstrated that analyses mainly based on the reporting of A(H7N9) cases, deaths or their demographics, such as epidemic curves in different regions, estimated onset-to-admission distributions, onset-to-death distributions and impact of poultry market closure can very closely match the results from official data sources with little time-lag. However, estimates of the fatality risk for hospitalized cases were less reliable based on public information, where the estimation requires follow-up of patient status after hospitalization. For example, there was a tendency for online news to highlight the first discharged case in each province but there were fewer reports on subsequent discharged cases. This is the first study to rigorously test the reliability of publicly available data for epidemiological purposes and, although the assessment of clinical severity may be limited, it shows the assessment of transmissibility and geographical dispersion to be reliable. Our results concur with a recent study of information on confirmed cases reported to the World Health Organization in the 2009 influenza pandemic, which also identified difficulties in estimating severity from such datasets [20,21].

The volume of online information about an epidemic is mostly driven by public interests and concern [22]. For an epidemic of a newly emerging or re-emerging disease, spread and severity of the diseases are of major public concern and, hence, information on case counts, severe or death cases are usually reported in more detail, especially when they are associated with a new location. In our study we also found that death dates were more frequently and accurately reported than discharge dates [see Additional file 3: Figure S2]. Information saturation also came into effect as the epidemic progressed [9], which may have resulted in decreasing accuracy and completeness of some variables. This is similar to the second wave of the influenza A(H1N1) pandemic during which there was disproportionately less media coverage even with a higher number of hospitalizations and deaths in some locations compared to the first wave [23].

In this study we did not attempt to estimate the incubation period, a potentially important epidemiological parameter for the control of disease transmission and for models of disease spread. The Virginia Tech line lists did collect information on occupational exposure, but more detailed individual information on poultry exposure was only available in the official line list. There was only limited information on poultry exposure for more severe cases in online news reports. Greater and more consistent details on the exposure history of individual cases, such as mode and different times of contact, are needed to allow robust analyses on the incubation period [4]. However, in a separate modeling study of the impact of live poultry market closures, we were able to obtain a reasonable estimate of the incubation period for A(H7N9) [15], and similar inference could be possible based on the publicly-available line lists.

There are several limitations in this study. The human influenza A(H7N9) epidemic in 2013 was mostly confined to the eastern part of China. Public data is likely to be less consistent, in terms of timeliness and accuracy, for diseases spreading across countries with different levels of healthcare resources, culture or local political environments. Secondly, duplicate reporting from different data sources may have inconsistent epidemiological information. National or international health organizations were regarded as most reliable but there were no well-defined rules for resolving inconsistencies. Thirdly, since current evidence shows that avian-to-human is the major transmission mode for influenza A(H7N9) [15,24], our analyses may not be directly generalizable to diseases with human-to-human transmission, especially those with such relatively high transmissibility that the scale may overwhelm official health authorities as in the A(H1N1) pandemic in 2009 to 2010. Monitoring the evolving transmissibility of emerging influenza viruses is crucial, but requires fairly accurate information about the onset of symptoms of the cases in addition to reliable exposure history information, and the understanding of the transmission dynamics among poultry and from poultry to humans. For the line lists using publicly available data this information is very limited, thus hindering quantification of transmissibility in terms of the basic reproduction number. Finally, there are diverse purposes for compiling different line lists. For example, the main purpose of HealthMap is to generate early outbreak notifications and map disease occurrences. Hence, by design that line list placed less emphasis on health status after hospitalization. The goal and methods of data collection can influence their ultimate utility.

For the specific purpose of epidemiological inference, only a minimal dataset with standardized format and definition [25], along with regular follow-up of patient status, may improve data accuracy, completeness and timeliness over the course of an epidemic. This essential dataset may avoid a too demanding requirement on data completeness at the expense of sustainability or accuracy, and help in reaching a consensus on the amount of details to be disclosed while maintaining appropriate patient confidentiality even in a public health emergency. For the MERS epidemic, the national health authorities of the affected countries have released information at different times and sometimes with very limited resolution [26,27], which would lead to challenges for any epidemiologist to unify all of the information into a single consistent database. In future emerging infectious disease outbreaks, depositing a line list into a database with agreed fields and hosted by a public platform, similar to the GISAID approach, and attaching corresponding time stamps and sources to each updated variable may also avoid confusion and improve accuracy.

## Conclusions

In conclusion, we have reported types of epidemiological inferences that can be reliably drawn from public information, and major limitations for assessment of clinical severity of the disease. As for the ongoing MERS epidemic and the return of influenza A(H7N9) in winter 2013 to 2014 (more than 200 new cases have been confirmed since October 2013) [28], a well-constructed line list will foster joint efforts for more timely analyses with broader perspectives. Our findings illustrate the increasing potential value of digital epidemiology or infoepidemiology, based on novel sources of information, such as social media, microblogs and mobile phone applications [9,29]. If publicly available information is sufficient to allow assessment of transmissibility and severity of emerging or reemerging infections [21,30], it may even be possible to crowdsource the analytical processes and obtain essential inferences more rapidly [31].

## Abbreviations

CDC: Center for Disease Control and Prevention; GISAID: The Global Initiative on Sharing All Influenza Data; HFR: hospitalization fatality risk; HKUSPH: the University of Hong Kong School of Public Health; MERS: Middle-East-Respiratory-Syndrome.

## Competing interests

BJC has received research funding from MedImmune Inc. and Sanofi Pasteur, and consults for Crucell NV; GML has received speaker honoraria from HSBC and CLSA; the other authors declare that they have no competing interests.

## Authors’ contributions

EHYL, GML, BJC and HY designed the study. EHYL, TKT and PW performed the analyses. JZ, QL, BL, JSB, SS, JYW, SRM, CR, HJ, YL, JY, QZ, ZC, FL, ZP and LF collected data. EHYL wrote the first draft and all authors contributed to review and revision of the report. EHYL and JZ contributed equally to this work. BJC and HY are guarantors. All authors read and approved the final manuscript.

## Pre-publication history

The pre-publication history for this paper can be accessed here:

http://www.biomedcentral.com/1741-7015/12/88/prepub

## Supplementary Material

Additional file 1 **Data file.** HealthMap, Virginia Tech, Bloomberg, HKUSPH, and FluTrackers line lists for dates where updates or historical archives were available, 5 April to 31, 2013. Click here for file

Additional file 2: Table S1 Sources of publicly available information for each line list. **Table S2.** Days required since 10 Apr to obtain robust estimates from different line lists, defined by coefficients of variation <30% comparing to the most updated estimates from the China CDC line list. Click here for file

Additional file 3: Figure S1 Proportion of A(H7N9) cases from the five line lists successfully matched to the official China CDC line list, 10 April to 31 May 2013. Cases were matched by age, sex, province and onset dates using more exact criteria in the first round of matching, followed by a second round of matching allowing for larger discrepancy and missing values. **Figure S2.** Proportion of missing demographic and epidemiological variables for A(H7N9) cases from the five line lists, 10 April to 31 May 2013. The denominators of missing hospitalization, death and discharge dates were the number of hospitalized, died and discharged patients matched to the official line list. **Figure S3.** Accuracy of demographic and epidemiological variables for Influenza A(H7N9) cases from the five line lists matched to the official line list, 10 April to 31 May 2013. Accuracy was defined to be exact for age, sex, province and severe cases. A two day discrepancy was allowed for onset, hospitalization, death and discharge dates. **Figure S4.** Epidemic curve by date of illness onset at four different dates of analysis from the five line lists, 15 February to 1 May 2013, overlaid with the epidemic curve based on the China CDC line list (black). **Figure S5.** Onset-to-hospitalization distribution, onset-to-death distribution and onset-to-discharge distribution estimated from data available on 10, 17, 24 April and 1, 2 May 2013. Darker colors represent estimates based on more recent data. **Figure S6.** Age, sex distributions and number of A(H7N9) cases in Shanghai, Zhejiang and Jiangsu, proportion of hospitalized and discharge cases from the five line lists, 10 April to 31 May 2013. The open dots show the median value. Rectangles show the lower and upper quartiles and vertical lines show the 5th to 95th percentiles. Click here for file
