# Supplementary material for: Accuracy of epidemiological inferences based on publicly available information: retrospective comparative analysis of line lists of human cases infected with influenza A(H7N9) in China
Source: BMC Med. 2014 May 28;12:88. doi: 10.1186/1741-7015-12-88 (PMC4066833; doi:10.1186/1741-7015-12-88)
Supplement: Additional file 2: Table S1 — Sources of publicly available information for each line list. Table S2. Days required since 10 Apr to obtain robust estimates from different line lists, defined by coefficients of variation <30% comparing to the most updated estimates from the China CDC line list. [file 1741-7015-12-88-S2.doc]

**Additional file 2**

**Table S1** Sources of publicly available information for each line list.

| **Sources** | **HealthMap** | **Virginia Tech** | **Bloomberg** | **HKU SPH** | **FluTrackers** |
| --- | --- | --- | --- | --- | --- |
|  |  |  |  |  |  |
| Chinese Ministry of Health website | × |  | × | × | × |
| Provincial Ministry of Health website | × |  | × | × | × |
| World Health Organization website | × | × | × |  |  |
| ProMED-mail | × |  |  | × |  |
| US CDC website | × | × |  |  |  |
| FluTrackers | × | × |  |  | - |
| Provincial Ministry of Health official microblogs |  |  | × |  |  |
| Chinese online news | × | × |  | × | × |
| Chinese blogs | × |  |  |  |  |

CDC, Centers for Disease Control and Prevention

**Table S2** Days required since Apr 10 to obtain robust estimates from different line lists, defined by coefficients of variation < 30% comparing to the most updated estimates from the China CDC line list.

| **Epidemiological parameters** | **China CDC** | **HealthMap** | **Virginia Tech** | **Bloomberg** | **HKU SPH** | **FluTrackers** |
| --- | --- | --- | --- | --- | --- | --- |
| **Median** |  |  |  |  |  |  |
| Onset to hospitalization | 0 | -^a^ | 0 | 0 | 0 | 0 |
| Onset to death | 19 | 16 | ≥17 | NA^b^ | 11 | 0 |
| Onset to discharge | 18 | -^a^ | -^a^ | NA^b^ | NA^b^ | NA^b^ |
| HFR_1_ | 22 | 35 | NA^b^ | NA^b^ | NA^b^ | 27 |
| HFR_2_ | 27 | -^a^ | -^a^ | NA^b^ | -^a^ | -^a^ |

CDC, Center for Disease Control and Prevention; HKUSPH, The University of Hong Kong School of Public Health; HRF, hospital fatality risk; ^a^Not enough information to calculate the parameter for the specific line list, ^b^Robust estimate was not yet obtained at the end of study.
