# Supplementary material for: Accuracy of epidemiological inferences based on publicly available information: retrospective comparative analysis of line lists of human cases infected with influenza A(H7N9) in China
Source: BMC Med. 2014 May 28;12:88. doi: 10.1186/1741-7015-12-88 (PMC4066833; doi:10.1186/1741-7015-12-88)
Supplement: Additional file 3: Figure S1 — Proportion of A(H7N9) cases from the five line lists successfully matched to the official China CDC line list, 10 April to 31 May 2013. Cases were matched by age, sex, province and onset dates using more exact criteria in the first round of matching, followed by a second round of matching allowing for larger discrepancy and missing values. Figure S2. Proportion of missing demographic and epidemiological variables for A(H7N9) cases from the five line lists, 10 April to 31 May 2013. The denominators of missing hospitalization, death and discharge dates were the number of hospitalized, died and discharged patients matched to the official line list. Figure S3. Accuracy of demographic and epidemiological variables for Influenza A(H7N9) cases from the five line lists matched to the official line list, 10 April to 31 May 2013. Accuracy was defined to be exact for age, sex, province and severe cases. A two day discrepancy was allowed for onset, hospitalization, death and discharge dates. Figure S4. Epidemic curve by date of illness onset at four different dates of analysis from the five line lists, 15 February to 1 May 2013, overlaid with the epidemic curve based on the China CDC line list (black). Figure S5. Onset-to-hospitalization distribution, onset-to-death distribution and onset-to-discharge distribution estimated from data available on 10, 17, 24 April and 1, 2 May 2013. Darker colors represent estimates based on more recent data. Figure S6. Age, sex distributions and number of A(H7N9) cases in Shanghai, Zhejiang and Jiangsu, proportion of hospitalized and discharge cases from the five line lists, 10 April to 31 May 2013. The open dots show the median value. Rectangles show the lower and upper quartiles and vertical lines show the 5th to 95th percentiles. [file 1741-7015-12-88-S3.pdf]

Introduction

We compared the five line lists based on publicly available information (HealthMap, Virginia Tech, Bloomberg, HKUSPH, FluTrackers) with the official China CDC line list, in terms of data accuracy, missingness and various epidemiological parameters in Figures S1-S6. Date of analysis refers to US local time for HealthMap, Virginia Tech and FluTrackers line lists, and China local time for China CDC, Bloomberg and HKUSPH line lists. Here we provided additional details of the figures.

In Figure S1, we matched the cases from the five line lists to the official China CDC line list. More exact criteria were applied in the first round of matching, allowing for missing values in one of the four matching variables. In the second round of matching the remaining cases were matched allowing for larger discrepancy and missing values in two of the four matching variables.

Some of the variables were not collected by the line lists. In Figure S2 and S3, HealthMap did not collect data on hospitalization admission and discharge dates. Virginia Tech, HKUSPH and FluTrackers did not collect data on discharge dates. In Figure S6, HealthMap did not collect data on hospitalization status and severe cases. Bloomberg did not collect data on severe cases.

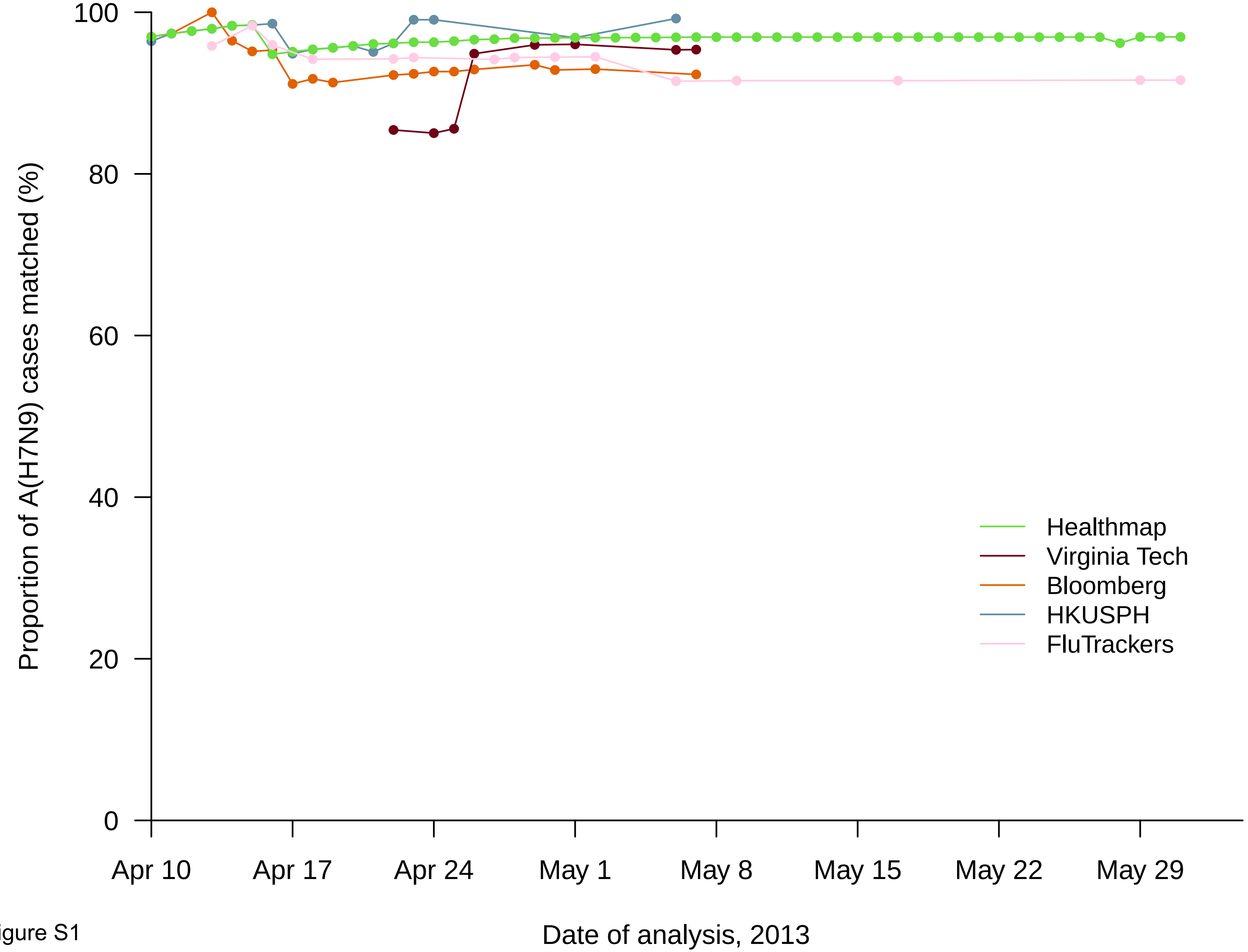

Proportion of A(H7N9) cases matched (%)
100
80
60
40
20
0
Apr 10
Apr 17
Apr 24
May 1
May 8
May 15
May 22
May 29
Date of analysis, 2013
Healthmap
Virginia Tech
Bloomberg
HKUSPH
FluTrackers

Figure S1

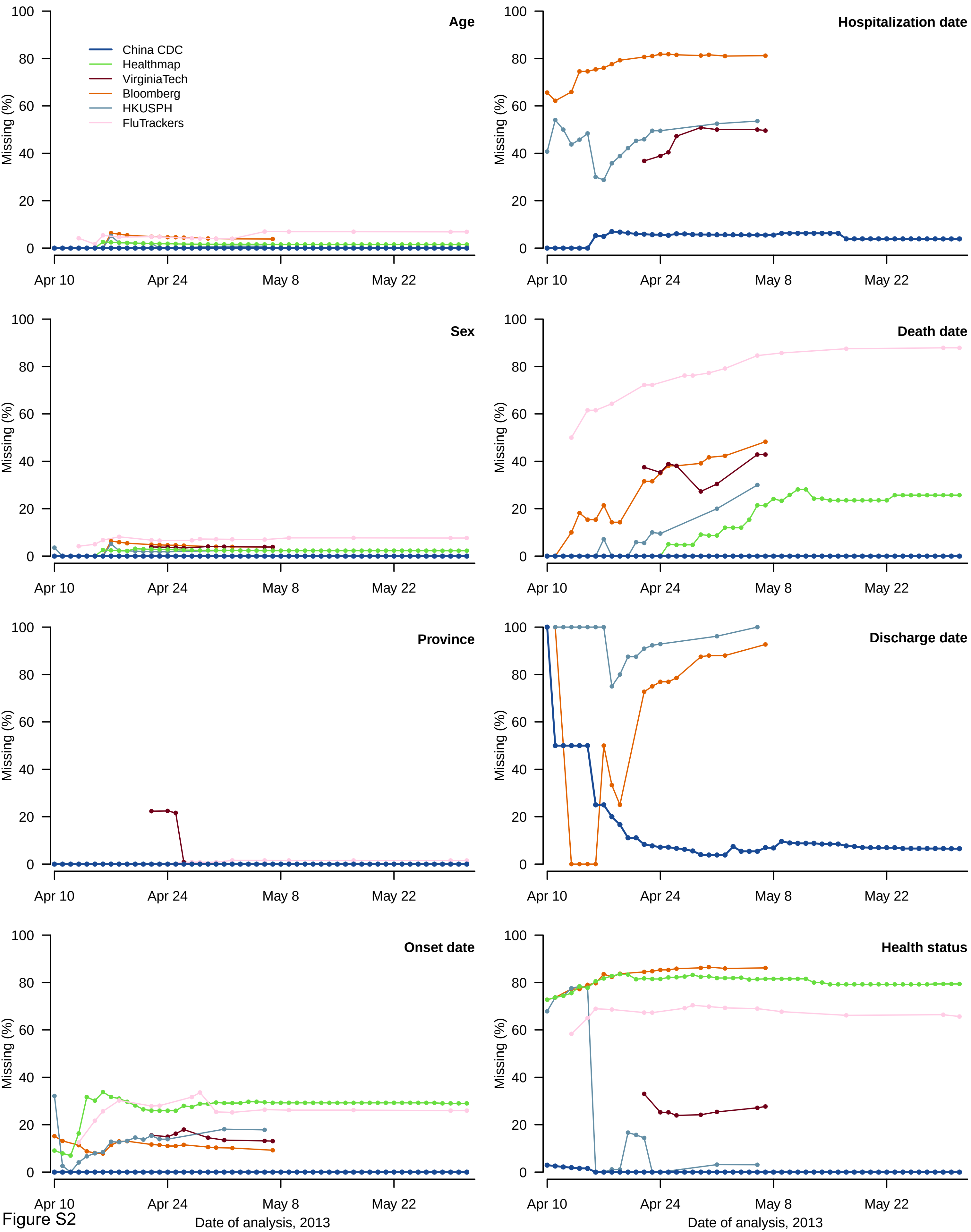

Age
China CDC
Healthmap
VirginiaTech
Bloomberg
HKUSPH
FluTrackers
Hospitalization date
Sex
Death date
Province
Discharge date
Onset date
Health status
Missing (%)
Apr 10
Apr 24
May 8
May 22
Date of analysis, 2013

Figure S2

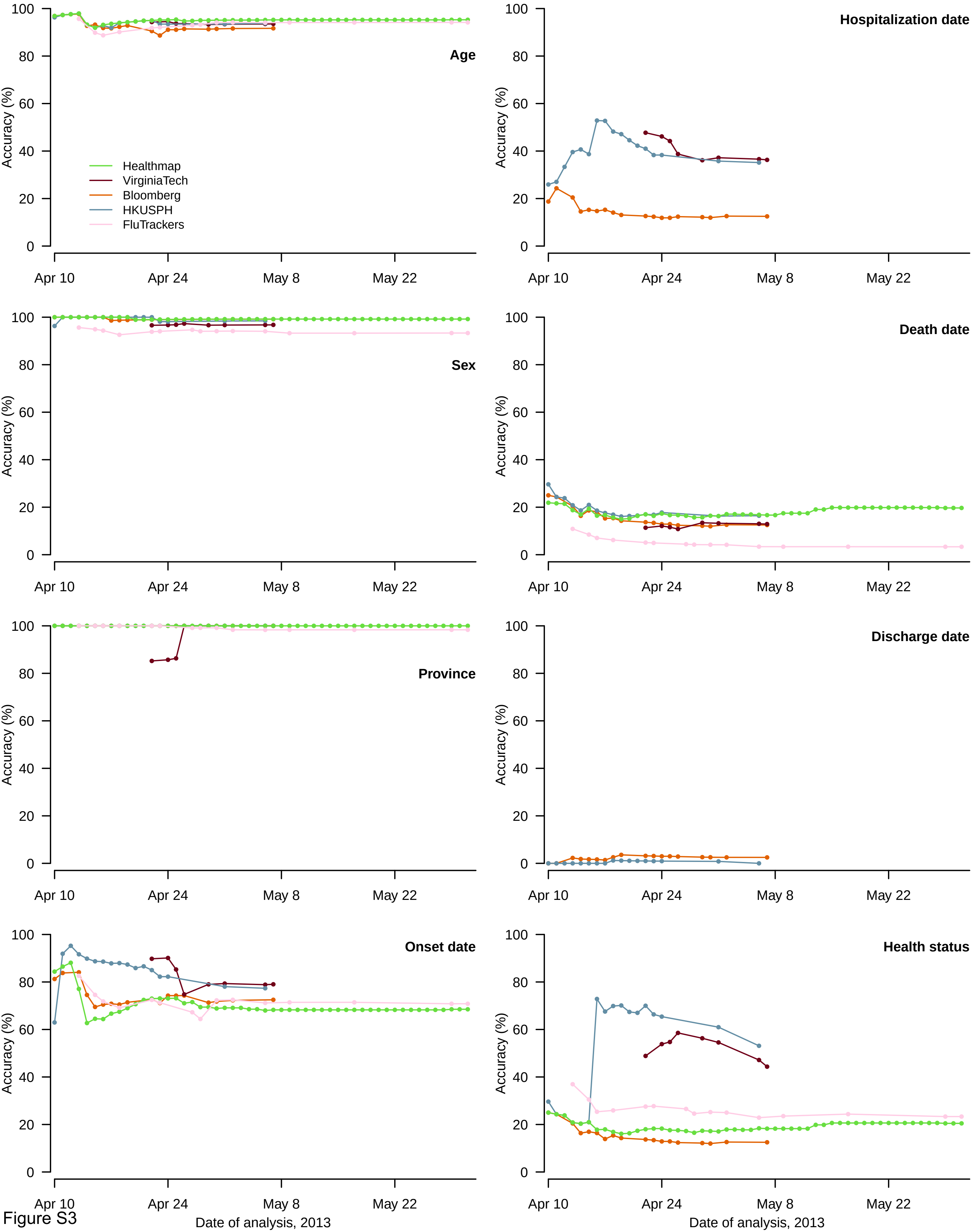
Age
Hospitalization date
Sex
Death date
Province
Discharge date
Onset date
Health status
Healthmap
VirginiaTech
Bloomberg
HKUSPH
FluTrackers
Accuracy (%)
Apr 10
Apr 24
May 8
May 22
Date of analysis, 2013
Figure S3

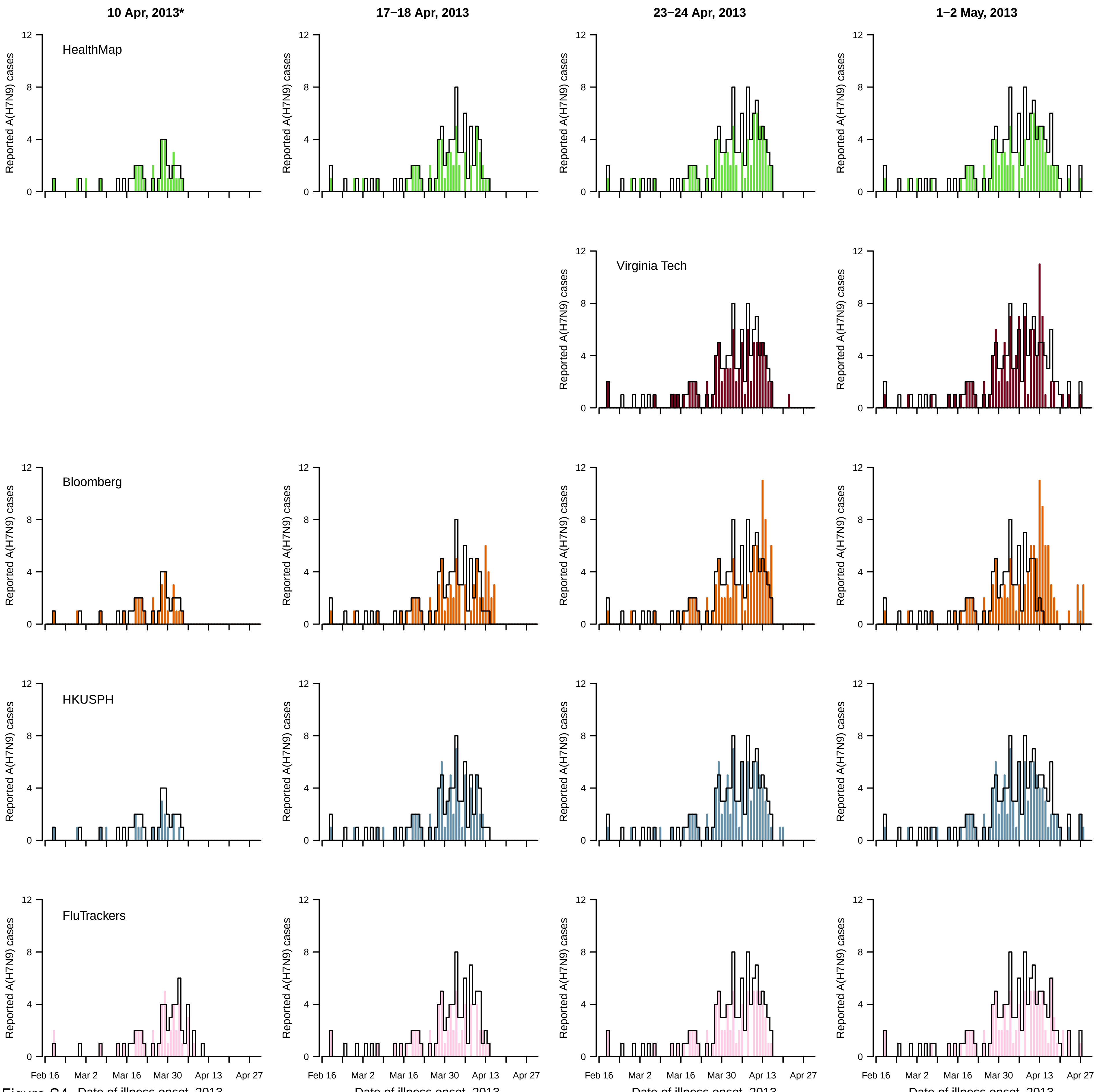
10 Apr, 2013*
17−18 Apr, 2013
23−24 Apr, 2013
1−2 May, 2013
HealthMap
Virginia Tech
Bloomberg
HKUSPH
FluTrackers
Reported A(H7N9) cases
0
4
8
12
Feb 16
Mar 2
Mar 16
Mar 30
Apr 13
Apr 27
Date of illness onset, 2013

Figure S4

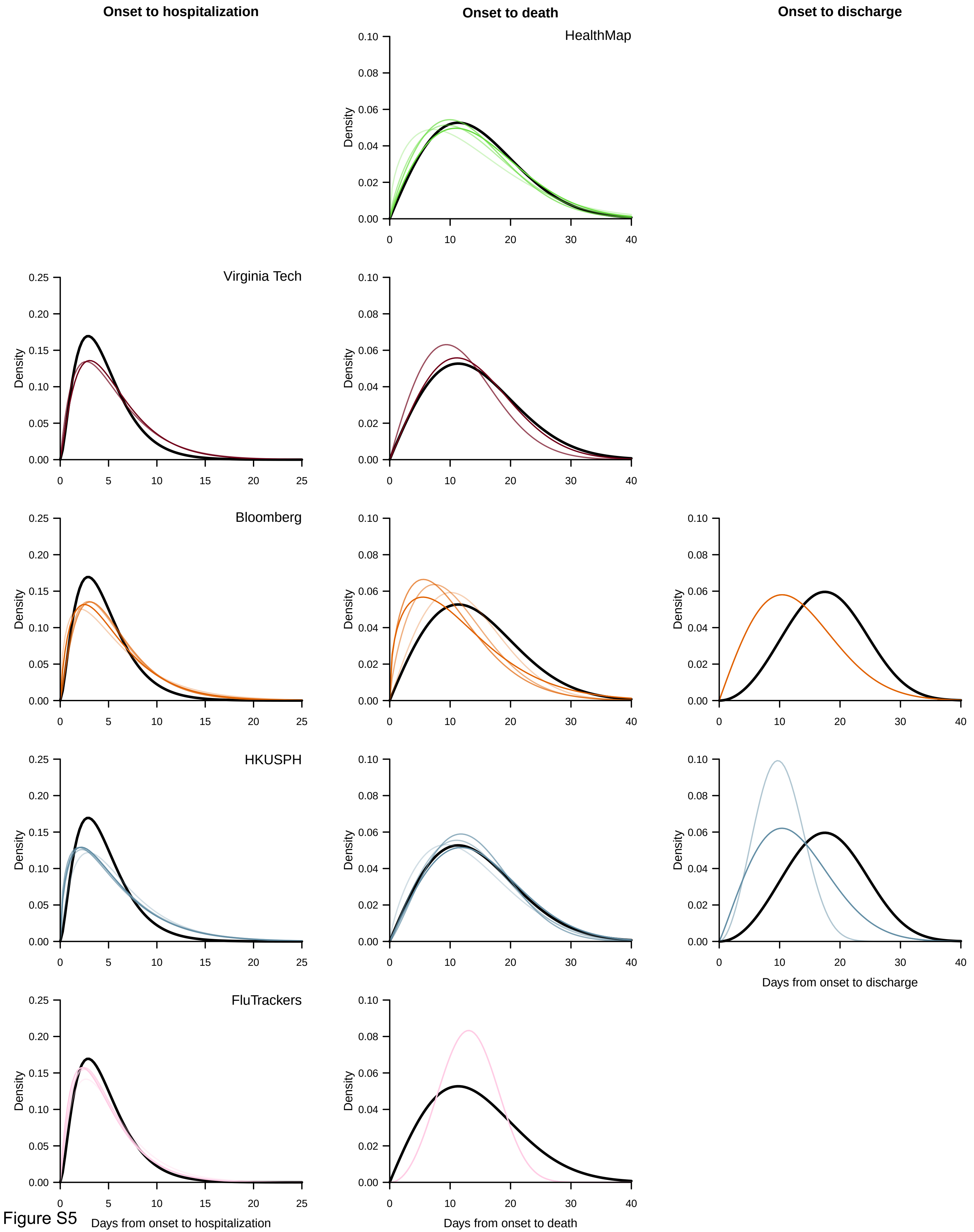

Onset to hospitalization
Onset to death
Onset to discharge
HealthMap
Virginia Tech
Bloomberg
HKUSPH
FluTrackers
Density
Days from onset to hospitalization
Days from onset to death
Days from onset to discharge

Figure S5

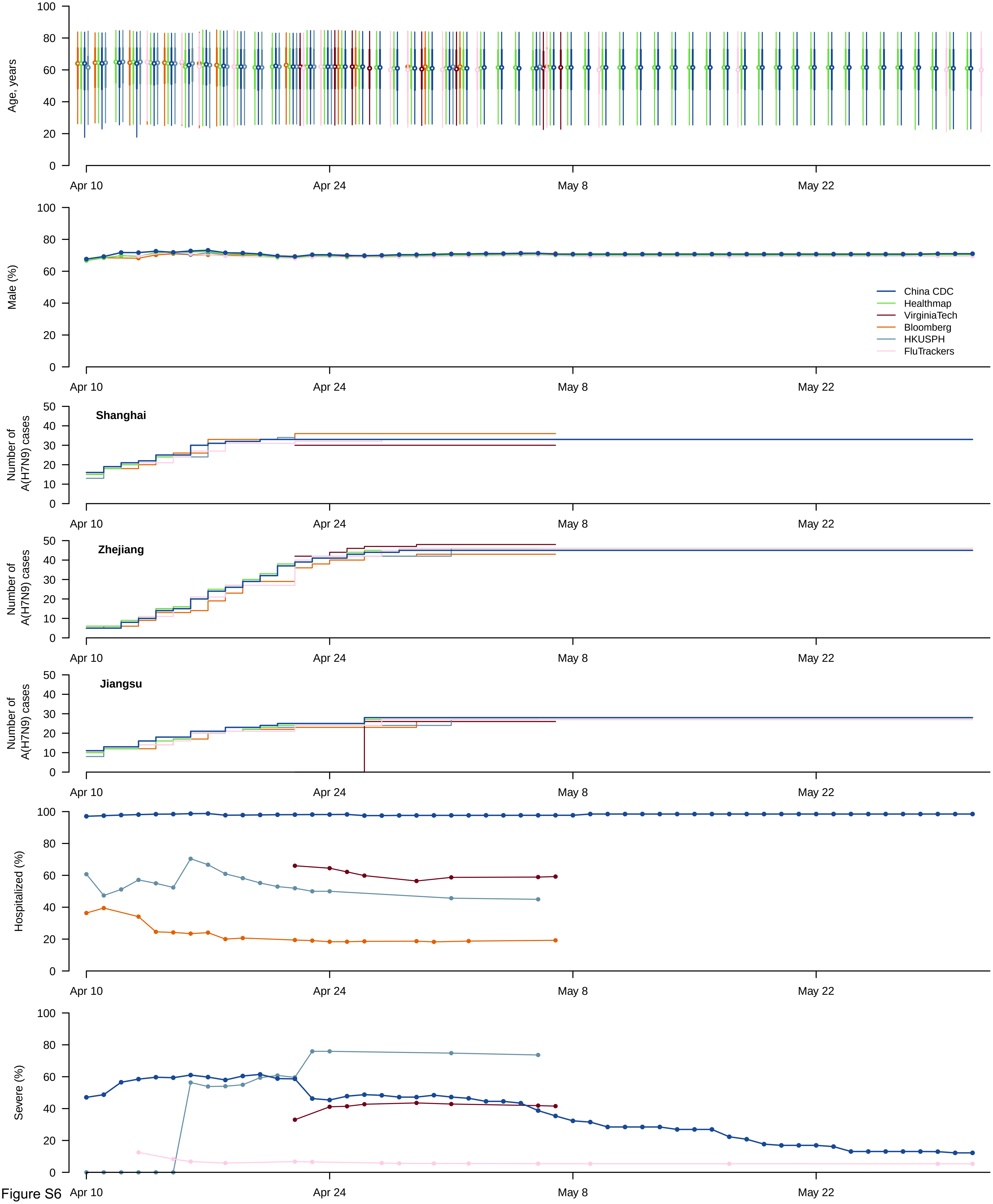

Age, years
Male (%)
China CDC
Healthmap
VirginiaTech
Bloomberg
HKUSPH
FluTrackers
Shanghai
Zhejiang
Jiangsu
Number of A(H7N9) cases
Hospitalized (%)
Severe (%)
Apr 10
Apr 24
May 8
May 22

Figure S6
